# Adherence to the Porto Criteria Based on the Hungarian Nationwide Pediatric Inflammatory Bowel Disease Registry (HUPIR)

**DOI:** 10.3389/fped.2021.710631

**Published:** 2021-09-03

**Authors:** Katalin E. Müller, Antal Dezsőfi, Áron Cseh, Dániel Szűcs, Noémi Vass, Éva Nemes, Orsolya Kadenczki, András Tárnok, Erzsébet Szakos, Ildikó Guthy, Márta Kovács, Anna Karoliny, Judit Czelecz, István Tokodi, Erika Tomsits, Gábor Veres

**Affiliations:** ^1^Heim Pál National Pediatric Institute, Budapest, Hungary; ^2^Institute of Translational Medicine, University of Pécs, Pécs, Hungary; ^3^Ist Department of Pediatrics, Semmelweis University, Budapest, Hungary; ^4^Department of Pediatrics, Szent-Györgyi Albert University, Szeged, Hungary; ^5^Department of Pediatrics, Clinical Center, University of Debrecen, Debrecen, Hungary; ^6^Department of Pediatrics, Medical School, University of Pécs, Pécs, Hungary; ^7^Borsod-Abaúj-Zemplén County Central University Hospital, University of Miskolc, Miskolc, Hungary; ^8^Jósa Hospital, Nyíregyháza, Hungary; ^9^Petz County Hospital, Győr, Hungary; ^10^Bethesda Children's Hospital, Budapest, Hungary; ^11^Szt. György Hospital, Székesfehérvár, Hungary; ^12^2nd Department of Pediatrics, Semmelweis University, Budapest, Hungary

**Keywords:** inflammatory bowel disease, children, diagnostic work up, Porto criteria, Crohn's disease, ulcerative colitis, MR enterography

## Abstract

**Objectives:** According to the Porto criteria, upper endoscopy and ileocolonoscopy with histology for patients with pediatric inflammatory bowel disease (pIBD) are recommended with small bowel imaging (SBI). We aimed to evaluate the adherence to the Porto criteria and biopsy sampling practice and to evaluate the diagnostic yield of magnetic resonance enterography (MRE) first time in a nationwide pIBD inception cohort.

**Methods:** Newly diagnosed pIBD cases (ages 0–18 years) are registered in the prospective, nationwide Hungarian Paediatric IBD Registry (HUPIR). We analyzed the diagnostic workup of patients recorded between the 1st of January 2007 and the 31st of December 2016.

**Results:** Data for diagnostic workup was available in 1,523 cases. Forty percent of the cases had complied with the Porto criteria. Adherence to the Porto criteria increased significantly from 20 to 57% (*p* < 0.0001) between 2007 and 2016. The most frequent reason for the incomplete diagnostic work-up was the lack of small bowel imaging (59%). In 2007, 8% of cases had a biopsy from all segments, and this rate reached 51% by 2016 (*p* < 0.0001). We analyzed the diagnostic yield of MRE in 113 patients (10.1%), who did not have any characteristic lesion for Crohn's disease. The MRE was positive for the small bowel in 44 cases (39%).

**Conclusions:** Adherence to the Porto criteria increased significantly during the 10-year period. This is the first study that reports multiple biopsy sampling as the less accepted recommendation. The diagnostic yield of MRE in patients without characteristic lesion for Crohn's disease is 39%.

## Introduction

In the last few decades, population-based registries revealed globally rising rates of inflammatory bowel disease (IBD) (1, 2). Pediatric onset-IBD (pIBD) occurs in 15–25% of all patients with IBD. The first epidemiological studies described some unique features of pIBD compared to adult IBD (3). However, comparison of these early studies was difficult because of their heterogeneity regarding diagnostic criteria, age-group and study design. In 2005, the IBD Working Group of the European Society for Pediatric Gastroenterology, Hepatology and Nutrition (ESPGHAN) published the Porto diagnostic criteria, a consensus guideline for the diagnosis of IBD to have consistent data on the epidemiology, phenotype and natural course of pIBD (4).

Based on the Porto criteria, all children with suspected IBD should undergo a complete diagnostic program including ileocolonoscopy, esophagogastroduodenoscopy (EGD), and small bowel imaging (SBI). Additionally, multiple biopsies from all gastrointestinal (GI) segments are necessary for a histological evaluation. To audit the Porto criteria, Eurokids Registry was launched to collect data on new pIBD patients. This registry has described an increase in the quality of diagnostic workup: adherence to the Porto criteria increased significantly from 45% in year 1 to 64% in year 5 (5). It is of note, that there is no detailed information about the histological sampling in pIBD.

Our aim was to analyze whether the diagnostic work-up changed during a 10-year long period based on the database of Hungarian Pediatric IBD Registry (HUPIR), furthermore, we also evaluated the diagnostic yield of magnetic resonance enterography (MRE) based on these real-life population data. In addition, this is the first report on the rate of multiple biopsy sampling in a nationwide pIBD cohort.

## Methods

On behalf of the Hungarian Pediatric Gastroenterology Society, a prospective registry of pIBD (HUPIR) was launched on the 1st of January 2007. Cooperation of 27 institutes has ensured the coverage of the whole country. Data collection has been described in detail previously (6). Questionnaires are filled out by gastroenterologists who made the diagnosis of IBD. Newly diagnosed IBD patients younger than 18 years are reported. Exclusion criteria are: age at diagnosis older than 18 years, and missing information on ileocolonoscopy and ileocolonic histology, and a diagnostic workup without endoscopic, histologic, and radiologic abnormalities and patients without informed consent. The questionnaires are collected via email and original data are validated by KEM and GV. Age, gender, familiarity (first-degree), disease location and behavior are recorded. Diagnostic procedures and their results including laboratory parameters, endoscopy, radiology, histology, surgical interventions are documented. The time of diagnosis was defined as the time of the endoscopic procedure and initiation of therapy for IBD. If the therapy was not initiated at the time of the endoscopy, then we defined the time of diagnosis when the treatment was started. The survey obtains the data anonymously. The study was approved by the National Ethical Committee.

We analyzed the diagnostic workup of patients recorded in the registry between the 1st of January 2007 and the 31st of December 2016. Altogether 1,624 patients were registered. However, 48 children were excluded, either by fulfilling the exclusion criteria, or because their diagnosis changed during the follow-up (e.g., irritable bowel syndrome, lymphoma, yersiniosis, etc.). Furthermore, in 53 cases the information about the diagnostic work-up was missing (due to surgery and delayed endoscopy or endoscopy performed in a non-participating adult center), therefore we were able to analyze the diagnostic work-up in 1,523 cases. Location and phenotype of pIBD were based on the Paris classification (7).

The workup in Crohn's disease (CD) and in patients with inflammatory bowel disease-unclassified (IBD-U) was considered complete when EGD, colonoscopy with ileal intubation, and adequate imaging of the small bowel were performed based on the Porto criteria (7). A colonoscopy was defined as a procedure used for reaching the cecum. In UC patients, a complete workup was defined as a combination of EGD and ileocolonoscopy. SBI was considered adequate when one of the following modalities was used: conventional radiology [small bowel follow-through (SBFT), enteroclysis], MRE, computed tomography (CT) scan, capsule endoscopy, and/or enteroscopy.

The Porto Group recommends multiple biopsies from each segment of the GI: esophagus, stomach, duodenum, terminal ileum (TI), cecum, ascending, transverse and descending colon, sigmoid and rectum. We also evaluated the adherence to the histological sampling recommendation.

Furthermore, we evaluated the diagnostic yield of MRE as the preferred SBI (8). Since macroscopic lesions of endoscopy were collected from 2010, we included children who were diagnosed after 1st January 2010. We analyzed the data of children without a characteristic lesion for CD based. Children were included into this sub-analysis, who qualified in the following criteria: (1) no aphtha, or serpiginous ulcer or pseudopolyp in the upper GI tract (characteristic for CD); (2) no ileoscopy or negative ileoscopy; (3) no perianal disease according to the Paris Classification; (4) no intraabdominal abscess, fistula or stricture; (5) no granuloma in histological results, but they had an MRE to evaluate the small bowels. We analyzed the MRE positivity of the small intestine and TI in these patients to evaluate the diagnostic yield of MRE.

Descriptive statistics were calculated as percentages for categorical data and medians and interquartile ranges for continuous variables. We compared continuous data where the Mann–Whitney *U*-test or Kruskal–Wallis test was used depending on the groups. A *p* < 0.05 result was considered as significant. Statistical analyses were performed using the SPSS (version 21.0; SPSS Inc., Chicago IL).

## Results

Between 1st of January 2007 and 31st December 2016, 1,576 children with newly diagnosed IBD were registered in HUPIR after validation. Diagnostic workup was not recorded completely in 53 cases (3%). Therefore, we analyzed the data of the remaining 1,523 patients; among them, 968 (63.6%) had CD, 474 (31.1%) had ulcerative colitis (UC) and 81 cases (5.3%) were diagnosed as IBD-U. Baseline clinical and demographic characteristics of the patients are shown in Supplementary Table 1.

### Evaluation of Diagnostic Workup Within the HUPIR Cohort Between 2007 and 2016

EGD was performed in 78.2% of all patients (1,191/1,523). The rate of EGD was significantly higher in CD (83.6%), than in UC (66.9%, *p* < 0.0001). In 71.9% of all patients where ileocolonoscopy was performed, the rate of ileocolonoscopy was significantly higher in CD patients than in UC cases (75.6 vs. 65.6%, *p* < 0.0001). Colonoscopy reaching the cecum was fulfilled in 89.5% of children. There was no significant difference in the subclasses of IBD. Adequate SBI was performed in 41.6% of newly diagnosed IBD patients, and the frequency of SBI was higher in CD (53.2%) and in IBD-U (51.9%), than in UC (16.0%) (CD vs. UC, *p* < 0.0001; IBD-U vs. UC *p* < 0.0001). The rate of each diagnostic procedure is shown in Table 1.

**Table 1:** Diagnostic workup of newly diagnosed IBD patients of HUPIR registered between 2007 and 2016.

	**Ulcerative colitis**	**Crohn's disease**	**IBD-U**	**All IBD**
Patient (*n*)	474	968	81	1523
EGD (%)	317 (66.9)	809 (83.6)^a^	62 (76.5)	1191 (78.2)
Ileocolonoscopy (%)	311 (65.6)	732 (75.6)^a^, ^b^	52 (64.2)	1095 (71.9)
Colonoscopy (%)	397 (83.8)	893 (92.3)^a^, ^b^	73 (90.1)	1363 (89.5)
SBI (%)	76 (16.0)^c^	515 (53.2)^a^	42 (51.9)	633 (41.6)
Histology from all segments (%)	140 (29.5)	288 (29.8)	29 (35.8)	457 (30.0)
Porto criteria (%)	235 (49.6)^c^	347 (35.8)^a^	27 (33.3)	609 (40.0)

*IBD, inflammatory bowel disease; IBD-U, inflammatory bowel disease type of unclassified; EGD, esophagogastroduodenoscopy; SBI, small bowel imaging.*

a *Significant difference compared to ulcerative colitis (p < 0.0001).*

b *Significant difference compared to IBD-U (p = 0.02).*

c *Significant difference compared to IBD-U (p < 0.0001)*.

### Adherence to the Porto Criteria Within the HUPIR Cohort Between 2007 and 2016

Thirty-six percent of children with CD (347/968), 33.3% of children with IBD-U (27/81) and 49.6% of UC patients (235/474) had complied with the requirements of the Porto criteria. Compliance in UC was significantly higher than in the other two IBD subgroups. However, only 54 out of 474 UC cases (11%) had EGD, ileocolonoscopy and adequate SBI. That was significantly lower than in CD (*p* < 0.0001) and IBD-U patients (*p* < 0.0001). In all subgroups the most frequent reason for the incomplete diagnostic workup was the lack of SBI.

We also applied a less strict analysis in CD and IBD-U, the work-up was regarded as completed when a child had either SBI or ileoscopy beside EGD and colonoscopy (up to the cecum). In this scenario 724 CD patients (75%) and 54 IBD-U patients (67%) had a complete work-up. Age, gender was not associated with the fulfillment of Porto criteria. Though, in case of positive family history rate of fulfilled Porto criteria were significantly higher (39.3 vs. 47.4%, *p* = 0.039).

### Time Trends of the Diagnostic Workup Within the HUPIR Cohort Between 2007 and 2016

**Figure 1:**
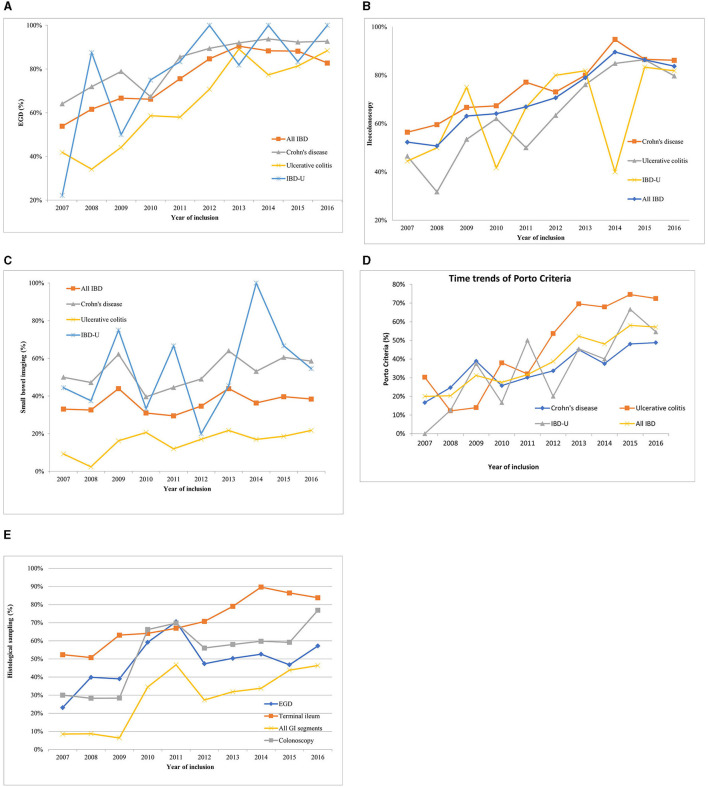
Time trends of diagnostic procedures within the HUPIR cohort from 2007 to 2016. **(A)** Time trends of esophagogastroduodenoscopy in ulcerative colitis, Crohn's disease and IBD-U between 2007 and 2016. **(B)** Time trends of ileocolonoscopy in ulcerative colitis, Crohn's disease and IBD-U between 2007 and 2016. **(C)** Time trends of adequate small bowel imaging in ulcerative colitis, Crohn's disease and IBD-U between 2007 and 2016. **(D)** Time trends of completed Porto criteria in ulcerative colitis, Crohn's disease and IBD-U between 2007 and 2016. **(E)** Time trends of histology sampling in paediatric inflammatory bowel disease between 2007 and 2016.

The frequency of EGD had shown a significant increase varying from 54 to 91% (*p* < 0.0001) in all types of IBD (CD: from 64 to 93%, *p* < 0.0001, UC: from 42 to 88%, *p* < 0.0001, IBD-U: 22 to 99%, *p* = 0.002) between 2007 and 2016 (Figure 1A). Ileoscopy has also become noticeably more frequent (from 52 to 84%) in all IBD patients (*p* < 0.0001). In CD, the rate of ileoscopy grew from 56 to 86% (*p* < 0.0001) over 10 years. Furthermore, a similar increase was observed in UC (from 47 to 80%, *p* < 0.0001) and in IBD-U (from 44 to 82%, *p* = 0.267) (Figure 1B).

**Figure 2:**
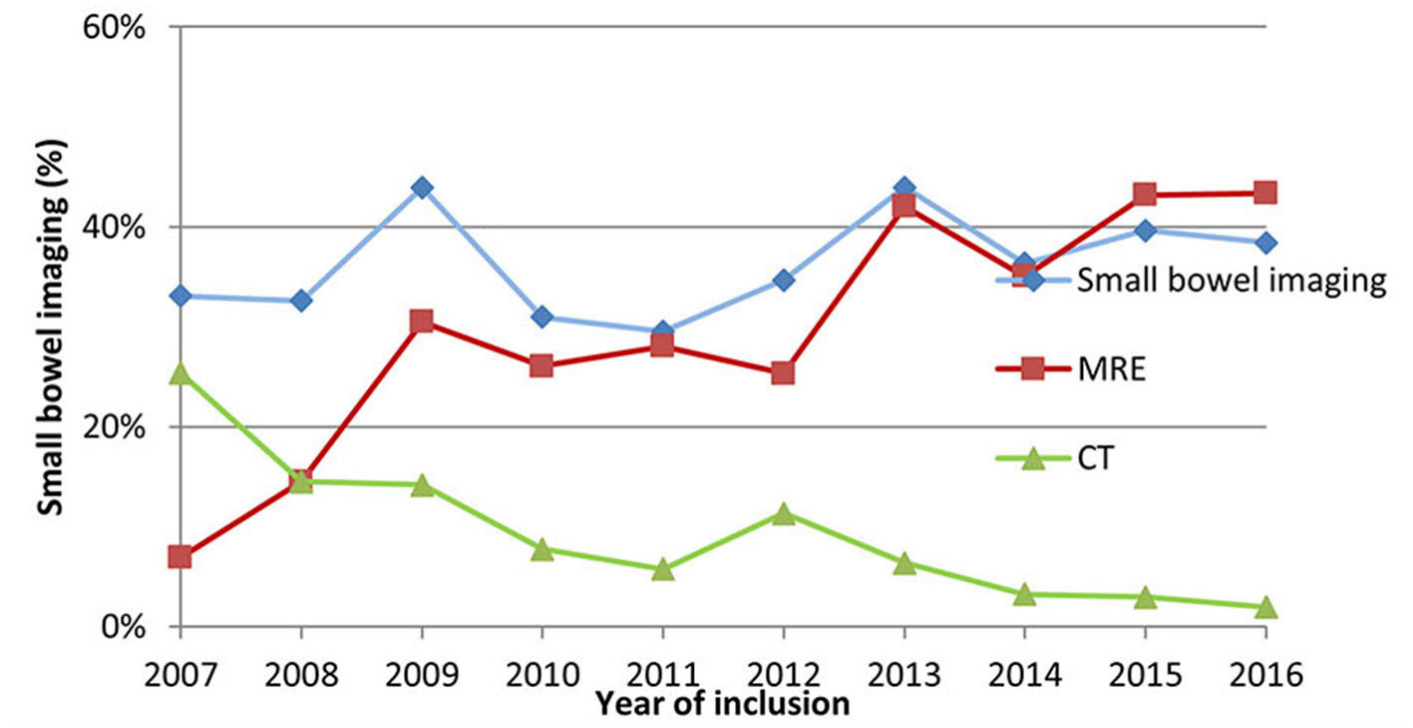
Rate of small bowel imaging, MRE and CT within the HUPIR cohort from 2007 to 2016.

Yet, the rate of adequate SBI in pIBD patients remained unchanged in this time-period (range 33–38%, *p* = 0.147) (Figure 1C). However, CT became rarer (from 25 to 2%, *p* < 0.0001), and MRE was performed more frequently (from 7 to 43%, *p* < 0.0001) (Figure 2). In 2016, 55% of CD children (69/114), 46% of IBD-U (6/13) and 22% of UC patients (15/68) had adequate SBI.

Adherence to the Porto criteria increased significantly from 20 to 57% (*p* < 0.0001) between 2007 and 2016 (Figure 1D). When analyzed by type of IBD, there was a notable time trend in adherence to the Porto criteria for patients diagnosed with CD (2007: 17%; 2016: 49%, *p* < 0.0001), for UC (2007: 30%; 2016: 72%, *p* < 0.0001), but not for IBD-U (2007: 0%; 2016: 55%, *p* = 0.282).

### Evaluation of Histological Sampling Practice

Multiple biopsies from all bowel segments are included in the Porto criteria. We found that only one third of CD and UC (CD: 29.8%, UC: 29.5%) patients and 35.8% of children with IBD-U had biopsies from each recommended GI segment. The highest adherence to multiple biopsies was observed in IBD-U, though the difference was not significant. Analyzing the histology sampling practice, we found that only 8% of newly diagnosed pIBD cases had a biopsy from all recommended segments (CD: 12%, UC: 5%, IBD-U:0%) in 2007. These rates grew remarkably by 2016 (8 vs. 51%, *p* < 0.0001). However, it is still not an everyday practice to take biopsies from all segments (Figure 1E). The low rate of multiple biopsy samples is due to the low sampling practice of EGD and colonoscopy [EGD: 63% (748/1,191), TI: 91% (991/1,095), colonoscopy: 61% (832/1,363)], and the difference was significant (EGD vs. TI, *p* < 0.0001, and TI vs. colonoscopy *p* = 0.0001).

### Diagnostic Yield of MRE

Adherence to the Porto criteria was not fulfilled mostly due to the lack of adequate SBI (Table 1). We analyzed the role of MRE in the diagnosis of IBD in a subgroup of patients recorded between 2010 and 2016. To evaluate the role of MRE 1,114 children were included, who were registered and validated between 2010 and 2016 (before 2010 we did not exactly record the endoscopic results). Among them, 113 patients (10.1%) fulfilled the criteria of “no characteristic lesion for CD.” The MRE was positive for the small bowel (jejunum or ileum and/or TI) in 44 cases (39%). According to these findings, the MRE was useful and important in establishing the diagnosis in 4% of patients (44/1,114), and in 39% of patients with colonic IBD and no characteristic sign of CD. From the 113 patients there were 72 children with negative ileoscopy. Among them, 15 (21%) had positive MRE (positive jejunum, ileum, and/or TI).

## Discussion

This is the first prospective nationwide pediatric IBD inception cohort registry that shows the diagnostic practice including histological sampling and its change during a 10-year long period. We found that the frequency of each diagnostic procedure, except SBI, increased. However, we detected an improvement in quality: frequency of MRE elevated in contrast to the decreasing CT-enterography. Furthermore, adherence to the Porto criteria has improved from 20 to 57% between 2007 and 2016. The acceptance of multiple biopsies has also changed. At the beginning of the registry <10% of the children had adequate histology sampling, which has improved (by 29%) over the 10 years period. Finally, we evaluated the diagnostic yield of MRE, and found that MRE was useful in 39% of patients without characteristic lesions of Crohn's disease.

De Bie et al. have presented the diagnostic workup of pIBD in Europe based on a prospective, web-based registry (Eurokids) reviewed between 2004 and 2009 (5). According to the analysis of 2,087 newly diagnosed IBD patients in the study of de Bie et al., EGD was performed in 87% of all pIBD patients, colonoscopy in 96%, and ileocolonoscopy in 72%. The rate of EGD in Hungary is a lower (78%), meanwhile the frequency of ileocolonoscopy is quite similar (72%). The diagnostic work-up in the last year (2016) was equal to the ones measured in the pIBD centers in Eurokids (2016: OGD: 83%, ileoscopy: 84%). This is remarkable, as it reflects the “average” practice of a whole country (large and small centers) compared to centers in Eurokids, that are committed to pIBD. The high rate of ileoscopy is similar to those of Eurokids and to the adult success rates (9), showing that the performing skills of Hungarian endoscopists are on a high level, and only the commitment of fulfilling ileoscopy and EGD could increase the rate of complete endoscopic workup.

Adequate SBI was performed in 53% of newly diagnosed pediatric CD and 52% of IBD-U patients. Interestingly, there was no significant time trend in this field. However, we detected an impressive decrease in CT, and an increase in MRE. Similarly, Buderus et al. also reported an increase in MRE rates from 19.4 to 56.5% between 2004 and 2014 based on the CEDATA registry, whereas they detected a sharp decrease in X-ray examinations, as we did in Hungary (10). It is of interest, that these SBI rates are much lower than what Eurokids reported in 2015 (CD: 72.5%, IBU-U: 61.9%) (11). In contrast to the rate of SBI, the rate of MRE was similar in HUPIR, Eurokids and CEDATA in CD (39 vs. 43 vs. 46%, respectively) and in IBD-U (40 vs. 27.9 vs. 20%) (10, 11). The difference reflects a distinctive approach; Hungarian pediatric gastroenterologists are less willing to perform other SBI modality if MRE is not available. It is also important, that we accepted MRE, in cases when it had been performed within 3 months of diagnosis, although waiting times can be longer in some centers in Hungary. Therefore, the rate of MRE (and by that the rate of the fulfilled Porto criteria) could be higher. It is of note, that there was a significant difference in the rate of MRE in different centers (tertiary vs. secondary centers, data not shown) reflecting the dissimilar availability of MRE.

Altogether the Porto criteria were fulfilled in 40% of newly diagnosed IBD patients, lower than the Eurokids cohort (2005–2013: 60%). Other population-based studies did not evaluate the changing trends in diagnostic work-up, except one study from the CEDATA registry (10). Supplementary Table 2 summarizes the data of some population-based studies that reported the rate of EGD, ileocolonoscopy and MRE. The comparison is difficult due to the heterogeneity of the studies. We can conclude that the Hungarian diagnostic practice was similar to the international data. During the study period (2007–2016) revised Porto criteria were published (June of 2014), and may influence our results. However, the tendencies did not show changes in the last 1.5 year.

Biopsies from all bowel segments are recommended according to the Porto criteria. We found this was the less accepted recommendation in Hungary. Biopsy from the TI is accepted (91%), but biopsies from all colon segments and from the upper GI tract is often missing. There are only few data in the literature for comparison. Winter et al. did not include histology in the definition of the complete Porto criteria, since there was a large number of patients without a complete set of biopsies (11). Sawczenko et al. reported that samples for histology were reported as not having been taken in 4% of IBD patients. However, they did not report whether this means no biopsy at all, or incomplete sampling (12). The reason for less frequent biopsy is probably that it is considered to elongate the time needed for an endoscopy, which would lead to the increased use of anesthetic drugs (costs, side effects, and effectiveness of endoscopic theater), and could place an unnecessary strain on pathologists (12). At present, histologic remission is a debatable treatment-target in either CD or UC, though the initial histological involvement of the GI tract could be beneficial in the assessment of prognosis and in evaluation the disease activity during the disease course (13, 14). These results may arise the question whether multiple histological sampling is equally important in different scenarios during the diagnosis of pIBD. Furthermore, it is a question whether multiple histological sampling is only a problem in Hungary, or it is also a problem in other countries. Low- or middle-income countries may need modification in the recommendation of histological sampling in order to optimally manage their resources (anesthesia, pathology).

Finally, the diagnostic yield of MRE was evaluated in this nationwide real-life pIBD cohort. MRE seems to be the rate limiting factor to complete the Porto criteria. Its role in the establishment of the diagnosis is not obvious since the diagnosis can be based on the endoscopy in most cases. MRE was positive in 39% of the cases without characteristic lesion for CD supporting the diagnosis of CD. Although this was only 4% of the whole cohort, suggesting that MRE is not overly important in the diagnosis. On the other hand, the use of MRE at diagnosis can help to evaluate the disease activity, disease extent in small bowel, extramural complications and extraintestinal manifestations.

Furthermore, transmural healing is an upcoming target in the treatment of IBD, which also contributes to the importance of an initial MRE (13, 15).

There are some limitations in this report. As it is widely known, the collection of pediatric cases over the age of 15–16 years is problematic for pIBD registries, as some of these patients are diagnosed in adult GI care. Another limitation of our study that the follow-up endoscopies (in case of surgery at diagnosis, or technical problems of ileal intubation) were not recorded, these data could improve the rate of fulfilled Porto criteria. Also, we could not evaluate the diagnostic yield of multiple biopsies because the quality of pathological evaluation largely differs throughout the country. Similarly, the protocols applied for MRE, and the practices followed by radiologists are also different, consequently the diagnostic yield of MRE may also differ center by center.

In summary, HUPIR is the first nationwide inception of a cohort pIBD reporting the diagnostic practice including histology. Based on data of HUPIR the diagnostic practice has improved in the last decade and came close to the practice of pIBD centers in Europe in recent years. Our data highlight the problems of diagnostic workup, that can be useful in countries where incidence of pIBD is increasing. MRE has an important role in the final diagnosis and classification in patients with colonic disease without characteristic lesions for CD. This is the first pIBD study reporting the practice of histology sampling in pIBD and showing the lack of adherence to recommendations.

## Data Availability Statement

The raw data supporting the conclusions of this article will be made available by the authors, without undue reservation.

## Ethics Statement

The studies involving human participants were reviewed and approved by Medical Research Council Mailing address: 7-8 Széchenyi István tér, Budapest, H-1051, Hungary. Written informed consent to participate in this study was provided by the participants' legal guardian.

## Author Contributions

KM contributed to the conception of the work, execution, analysis, and interpretation of data. GV contributed to the conception of the work, interpretation of data, and wrote the final version of the paper. ÁC contributed to analysis and execution. AD participated in study design, manuscript drafting, and critical discussion. OK participated as clinical consultant and contributed to execution. EN participated as clinical consultant and contributed to execution. AT participated in study execution, manuscript drafting, and critical discussion. ES participated as clinical consultant and contributed to execution. MK participated as clinical consultant and contributed to execution. AK participated as clinical consultant and contributed to execution. JC participated as clinical consultant and contributed to execution. IT participated as clinical consultant and contributed to execution. ET participated as clinical consultant and contributed to execution. All authors contributed to the article and approved the submitted version.

## Conflict of Interest

The authors declare that the research was conducted in the absence of any commercial or financial relationships that could be construed as a potential conflict of interest.

## Publisher's Note

All claims expressed in this article are solely those of the authors and do not necessarily represent those of their affiliated organizations, or those of the publisher, the editors and the reviewers. Any product that may be evaluated in this article, or claim that may be made by its manufacturer, is not guaranteed or endorsed by the publisher.
