# Supplementary material for: Adherence to the Porto Criteria Based on the Hungarian Nationwide Pediatric Inflammatory Bowel Disease Registry (HUPIR)
Source: Front Pediatr. 2021 Sep 3;9:710631. doi: 10.3389/fped.2021.710631 (PMC8494028; doi:10.3389/fped.2021.710631)
Supplement: Supplementary file 1 [file Table_1.docx]

Supplementary Material

# Supplementary Table 1.

Basic demographic and disease specific characteristics of newly diagnosed IBD patients of HUPIR registered between 2007 and 2016

|  | Crohn's disease | Ulcerative colitis | IBD-U |
| --- | --- | --- | --- |
|  |  |  |  |
| No. (%) | 968 (63.6) | 474 (31.1) | 81 (5.3) |
| Male, No. (%) | 563 (58.7%)^a^ | 248 (52.3%) | 37 (45.7)^b^ |
| Age at diagnosis (median, IQR) (yr) | 14.3 (11.8-16.2)^a^ | 13.4 (10.5-15.6) | 13.5 (10.1-15.7)^b^ |
| Positive family history No. (%) | 130 (14.3) | 51 (11.7) | 10 (13.9) |
| CD location, No. (%) |  |  |  |
| Ileal, L1 | 177 (22%) |  |  |
| Colonic, L2 | 133 (16.4%) |  |  |
| Ileocolonic, L3 | 486 (60%) |  |  |
| Isolated upper tract, L4 | 13 (1.6%) |  |  |
| Upper tract | 404 (50%) |  |  |
| CD behaviour, No. (%) |  |  |  |
| Nonstricturing, B1 | 876 (90.5%) |  |  |
| Stricturing, B2 | 68 (7%) |  |  |
| Penetrating, B3 | 24 (2.5%) |  |  |
| Perianal disaease, P | 64/36 (10%) |  |  |
| UC extent, No. (%) |  |  |  |
| Proctitis, E1 |  | 38 (9%) |  |
| Left-sided, E2 |  | 112 (26%) |  |
| Extensive, E3 |  | 47 (11%) |  |
| Pancolitis, E4 |  | 237 (54%) |  |

^a^ CD data significantly different from UC (p<0.0001)

^b^ IBD-U data significantly different from CD (UC (p<0.0001))

CD: Crohn’s disease; UC: ulcerative colitis; IBD-U, inflammatory bowel disease type of unclassified; IQR: interquartile range
