# Supplementary material for: Adherence to the Porto Criteria Based on the Hungarian Nationwide Pediatric Inflammatory Bowel Disease Registry (HUPIR)
Source: Front Pediatr. 2021 Sep 3;9:710631. doi: 10.3389/fped.2021.710631 (PMC8494028; doi:10.3389/fped.2021.710631)
Supplement: Supplementary file 2 [file Table_2.docx]

Table 2. Diagnostic workup and Porto criteria in different cohort of pediatric inflammatory bowel disease

| Country  (reported years) | Diagnosis,  number of patients | EGD | Ileocolonoscopy | MRE | Porto Criteria |
| --- | --- | --- | --- | --- | --- |
| Hungary  (2007-2016) * | IBD, n=1523 | 78% | 72% | 31% | 66.5% |
|  | CD, n=968 | 84% | 76% | 39% | 36% |
|  | IBD-U, n=81 | 77% | 64% | 40% | 33% |
|  | UC, n=474 | 67% | 66% | 13% | 50% |
| EUROKIDS  (2005-2013)(11) | IBD, n=3461 | 89% | 78% | 33% | 60% |
|  | CD, n=2085 | 93% | 78% | 43% | 60% |
|  | IBD-U, n=265 | 88% | 76% | 28% | 48% |
|  | UC, n=1111 | 83% | 78% | 16% | 64% |
| Belgium, BELCRO cohort  (2008-2010)(16) | CD, n=255 | 75% | 85% | 13% | 38% |
| Slovenia (2002-2010)(17) | CD, n=167 | 93% | 90% | 10% |  |
|  | UC, n =105 | 77% | 86% | 2% |  |
| Germany and Austria,  CEDATA  (2004-2010, 2013-2014)(10) | CD, n=616 | 88% | 78% | 47% |  |
|  | UC, n=278 | 60% | 63% | 12% |  |
|  | IBD-U, n=64 | 69% | 69% | 20% |  |

IBD, inflammatory bowel disease;

CD, Crohn’s disease

UC, ulcerative colitis

IBD-U, inflammatory bowel disease type of unclassified;

EGD, esophagogastroduodenoscopy

MRE, MR enterography
